# Fetal cardiac cine magnetic resonance imaging *in utero*

**DOI:** 10.1038/s41598-017-15701-1

**Published:** 2017-11-14

**Authors:** Jerome Chaptinel, Jerome Yerly, Yvan Mivelaz, Milan Prsa, Leonor Alamo, Yvan Vial, Gregoire Berchier, Chantal Rohner, François Gudinchet, Matthias Stuber

**Affiliations:** 1 Department of Radiology, University Hospital (CHUV) and University of Lausanne (UNIL), Lausanne, Switzerland; 2 Center for Biomedical Imaging (CIBM), Lausanne, Switzerland; 3 Division of Pediatric Cardiology, Department Woman-Mother-Child, University Hospital (CHUV) and University of Lausanne (UNIL), Lausanne, Switzerland; 4 Division of Obstetrics and Gynecology, Department Woman-Mother-Child, University Hospital (CHUV) and University of Lausanne (UNIL), Lausanne, Switzerland

## Abstract

Fast magnetic resonance imaging (MRI) led to the emergence of ‘cine MRI’ techniques, which enable the visualization of the beating heart and the assessment of cardiac morphology and dynamics. However, established cine MRI methods are not suitable for fetal heart imaging *in utero*, where anatomical structures are considerably smaller and recording an electrocardiogram signal for synchronizing MRI data acquisition is difficult. Here we present a framework to overcome these challenges. We use methods for image acquisition and reconstruction that robustly produce images with sufficient spatial and temporal resolution to detect the heart contractions of the fetus, enabling a retrospective gating of the images and thus the generation of images of the beating heart. To underline the potential of our approach, we acquired *in utero* images in six pregnant patients and compared these with their echocardiograms. We found good agreement in terms of diameter and area measurements, and low inter- and intra- observer variability. These results establish MRI as a reliable modality for fetal cardiac imaging, with a substantial potential for prenatal evaluation of congenital heart defects.

## Introduction

During the past three decades, magnetic resonance imaging (MRI) has been developed into a versatile modality for studying the physiology and pathology of the human cardiovascular system^1,2^. Unique windows into cardiac anatomy, function and hemodynamics have been opened up, in particular through the ability to acquire detailed static images along oblique axes as well as series of images that can be composed into video sequences, in an approach known as ‘cine MRI’. Moreover, today powerful techniques are available for quantifying blood flow and tissue perfusion. However, whereas cardiovascular MRI has become a part of the clinical imaging portfolio for adult and pediatric patients in the Western world^3,4^, the approach reaches its technological limits when it comes to assessing the condition of the fetal heart.

Cardiac imaging of the fetus is particularly important in screening for significant congenital heart defects (CHDs), which occur at an incidence of about 6–19/1000 live births worldwide^5^. Currently, prenatal screening for CHDs is performed with ultrasonography^6^. However, the sensitivity of fetal echocardiography varies widely, depending on equipment, national screening policies, level of training, examination practice and the population screened^7^. As a result, in many cases, CHDs remain undetected until birth. For example, in the US the rate of prenatal detection of CHDs requiring surgery (~25% of all CHDs) is only 42%^8^. Moreover, the evaluation of complex CHDs would benefit from a more precise delineation of their morpholgy, especially in cases where ultrasonographic methods reach their limits, such as in late gestational age, in cases of suboptimal fetal position or maternal habitus^9^, multiple gestations, oligohydramnios^10^, or poor ultrasonographic windows. Under these circumstances MRI-based methods promise a distinct advantage, given their relatively high spatial resolution and independence from specific access windows.

However, since the first attempts more than two decades ago^11^, technical developments in the area of fetal cardiac MRI have remained relatively modest, preventing its translation into clinical routine^12–14^. Three major challenges are to be overcome: first, the absence of a readily accessible electrocardiogram (ECG) signal from the fetus to synchronize data acquisition; second, the unpredictable motion of the fetus; and third, the reduced anatomical size of the fetal heart^15^. So far, cardiac MRI studies of the fetus mostly relied on static imaging techniques^16^ or untriggered fast acquisition methods (typically based on real-time balanced steady-state free-precession sequences^17^) developed for organs other than the fetal heart^12–14^. However, none of these approaches are fully adequate since static imaging does not allow for functional assessment of the fetal heart (eg. cardiac contractility and valve function^18^), and untriggered acquisitions lack spatial and temporal resolution to address the above-mentioned challenges.

During the past few years, attempts have been made to obtain a triggering signal using external devices, such as MR-compatible cardiotocographs^19,20^ or electrodes^21^. However, cardiotocograph devices have to be appropriately positioned on the mother’s abdomen, and MR-compatible devices have so far not been tested in humans. Electrode-based devices, on the other hand, can also interfere with the gradient magnetic fields needed for MRI and lead to mistriggering^21^. A more promising solution to overcome the absence of a fetal ECG signal is cardiac self-gating^22^. Instead of using an external device to record the ECG signal, self-gating approaches are based on detecting heart contractions directly from the imaging data. Two self-gating approaches have been explored in the context of fetal cardiac imaging. Yamamura *et al*.^23^ proposed the detection of the heart contraction of an ewe’s fetus by analyzing the modulation of the signal amplitude of the *k*-space center, making the assumption that this signal is linked with the fetus’ cardiac cycle. However, considering the relatively small size of the fetal heart with respect to the large field of view, the signal-intensity variations induced by cardiac contraction might be challenging to detect. The second approach, Metric Optimized Gating^24^, was tested in human fetuses. It is based on iteratively estimating the fetus’ ECG through minimizing an image-entropy-based metric that is correlated with ghosting-reconstruction artifacts. The algorithm performs an exhaustive search of all possible combinations of readouts and selects the combination yielding the minimum entropy as the final self-retro-gated cine images. Whereas this technique was successfully tested in humans, image reconstruction is time consuming and sensitive to unpredictable fetal bulk motion.

Here we introduce a novel self-gating approach for fetal cardiac MRI and demonstrate that this technique delivers the spatial and temporal resolution needed for reliably identifying key anatomical structures used routinely in diagnoses of the fetal heart. The method builds on tools that we have recently developed in the context of assessing coronary endothelial function in adults^25^. We first reconstruct real-time images of limited image quality but high temporal resolution. These images are then analyzed to extract a self-gating signal that characterizes the periodic contraction of the fetal heart, which in turn is used to retrospectively sort all readouts into their corresponding cardiac phase. This framework enables the reconstruction of retrospectively self-gated cardiac cine images of high quality with no external ECG signal required for gating the acquisition. In a study including six pregnant patients who underwent both MRI and ultrasonography, we established the feasibility of cardiac fetal MRI in humans using this novel framework and the potential clinical value of the images obtained.

## Data-driven self-gating

Our method is based on first reconstructing and analyzing real-time images to extract a data-driven self-gating signal that characterizes the periodic contraction of the fetal heart. Data were collected using a continuous 2D slice-selective acquisition with a balanced steady-state free-precession sequence that includes a golden-angle radial trajectory. Such a trajectory ensures a relatively uniform coverage of *k*-space for any subset of radial profiles, thus enabling flexible reconstruction of real-time and retrospectively reordered images with arbitrary temporal resolution^26^.

**Figure 1:**
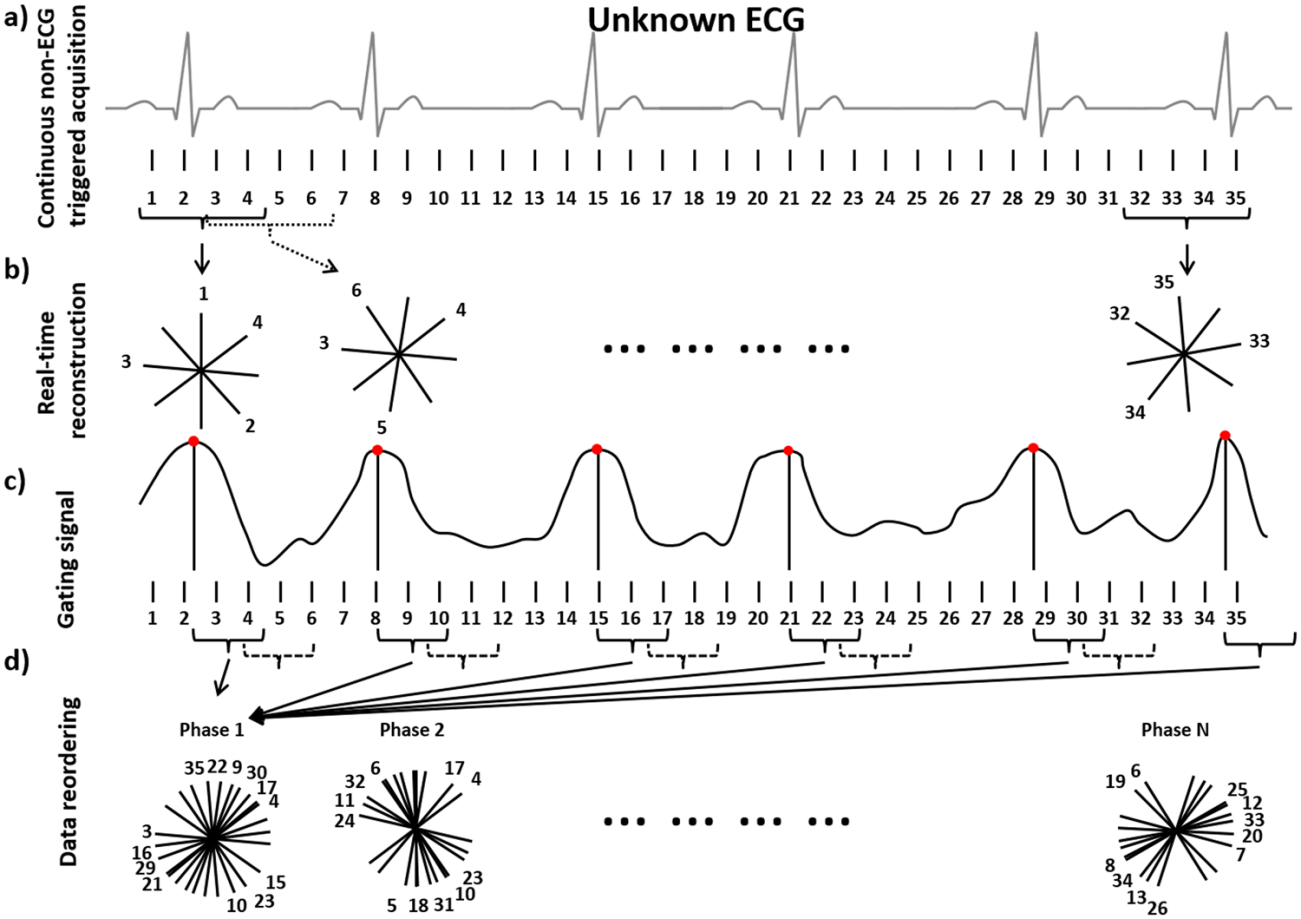
Schematic of the reconstruction framework. The data are acquired continuously irrespective of the cardiac cycle (**a**), and are reconstructed in real-time as a first pass (**b**). From the real-time images, the contraction of the fetal heart can be detected and a gating signal extracted (**c**). Using the gating signal, data are reordered according to the cardiac phase during which they were acquired, and images of high temporal and spatial resolution are reconstructed during a second pass (**d**).

Due to the high fetal heart rate (typically between 110 and 160 bpm), real-time images with high temporal resolution are required for accurately and precisely estimating the cardiac phases. In our protocol, these images are reconstructed off-line (see Methods) and analyzed semi-automatically to extract a self-gating signal (for details, see the Methods section). Two reference images corresponding to systole and diastole are manually identified in the real-time image series during a period when the fetus does not move. Subsequently, the operator defines a region of interest (ROI) at the location of the fetus’ heart. The ROI is then used to compute a cross-correlation signal for systole and diastole through the entire real-time image series, using the Pearson correlation coefficient (see gating signal, Fig. 1c). The two computed signals oscillate out of phase in a regular manner when the fetus does not move during acquisition. The signal with the steepest variations is then used as a gating signal. The peaks of the correlation signal indicate the highest similarity with the frame of reference (red dots, Fig. 1c). These peaks are manually selected by the operator and are used as trigger points to assign a cardiac phase to each of the readouts. Each radial readout is therefore time-stamped, based on the time delay from the prior trigger. The data are then retrospectively binned into different cardiac phases according to their time stamp, as in standard ECG retro-gated cine sequences (Fig. 1d, data reordering).

In our experiments, a bin width of 25 ms was used with data sharing of 50% between consecutive bins, leading to a temporal resolution of 12.5 ms. The self-retro-gated cine images were reconstructed with the same *k*-*t* Sparse SENSE algorithm used for reconstruction of the real-time images (see Methods). In the case of fetal motion at some point during acquisition, the amplitude of the correlation signals decreased significantly, which could easily be identified. In such cases, motion-corrupted data were rejected, excluded from the reconstruction, and did not contribute to the final images. In practice, this strategy had little adverse impact on image quality due to the resilience of the l_1_-constrained reconstruction and the properties of the radial golden-angle trajectory.

## *In utero* fetal cardiac MRI and comparison with ultrasonography

We imaged six pregnant patients (29.7 ± 2.1 weeks of pregnancy at the time of fetal MRI), referred initially for non-cardiac fetal MRI. MR cardiac cine data sets were always acquired after the clinically indicated exam (see Methods).

To assess both the performance and reliability of our approach, a comparison with echocardiography, the current gold standard, was performed. Echocardiographic cine images were acquired within a week of the MR examination (see Methods). Three standard views — four-chamber, three-vessel and short-axis — as commonly used in standard clinical fetal echocardiographic examinations^27^ were chosen to assess fetal cardiac anatomy and function in the MR images.

For a qualitative assessment on both MR and echocardiography, two experienced pediatric cardiologists independently evaluated the visibility and delineation of 13 anatomical structures of the fetal heart on a binary scale (1: visible, 0: not visible), using the following landmarks:

1. Four-chamber view: left and right atria, tricuspid and mitral valve annuli, left and right ventricles, the moderator band, and atrial and ventricular septa;
2. Three-vessel view: aorta, pulmonary artery, superior vena cava;
3. Short-axis view: left ventricular (LV) papillary muscles.

Quantitative comparison of the two imaging modalities was performed by measuring

1. the LV end-diastolic and end-systolic areas on the short-axis view;
2. the diameter of the tricuspid valve annulus;
3. the diameter of the mitral valve annulus;
4. the diameter of the aorta;
5. the diameter of the main pulmonary artery.

Measurements were performed using ClearCanvas (Synaptive Medical, Toronto, Canada). The two readers individually selected the most suitable images among all the images acquired during the examination. Measurements from both modalities were compared for the two observers in order to assess the reliability of MRI relative to echocardiography. An inter-observer variability analysis for both MRI and echocardiography was performed for the two observers. Intra-observer variability was assessed for the first observer, who repeated the measurements after more than three weeks on the same images as those analyzed during the first session.

Statistical analysis for quantitative measurements were performed using a paired two-tailed Student’s t-test (in which p < 0.05 was considered statistically significant), plus Bland–Altman analyses and linear regressions.

## Imaging results

**Figure 2:**
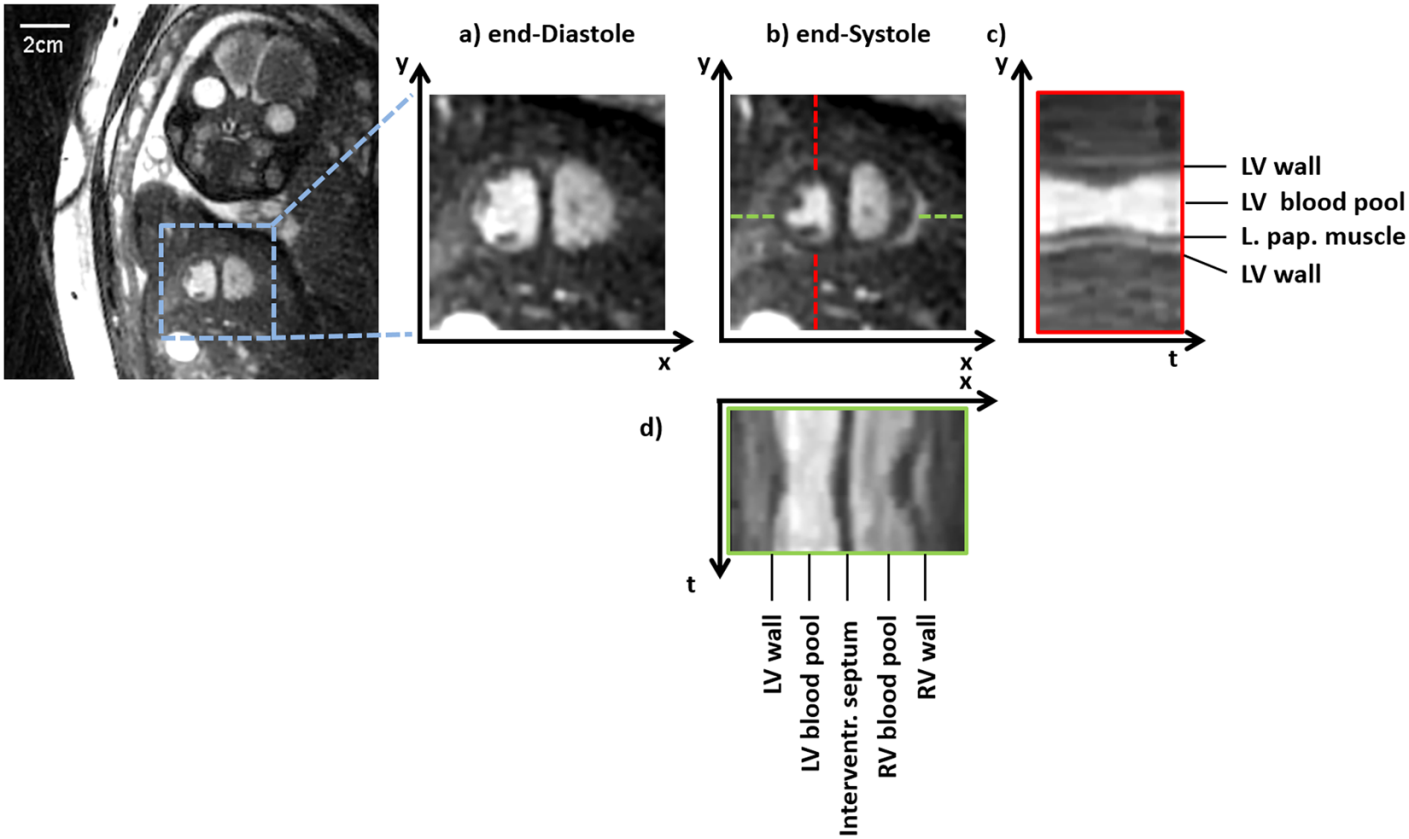
Self-retro gated reconstruction in a 28-week-old fetus in the short-axis view. Magnified views of the heart are shown in diastole (**a**) and systole (**b**), along with y–t space (**c**) and x–t space (**d**) traces obtained from the middle of the ventricles. The contraction of the heart and the thickening of the myocardium can be seen in both x–t and y–t space. For the corresponding movie, see the Supplementary Information.

**Figure 3:**
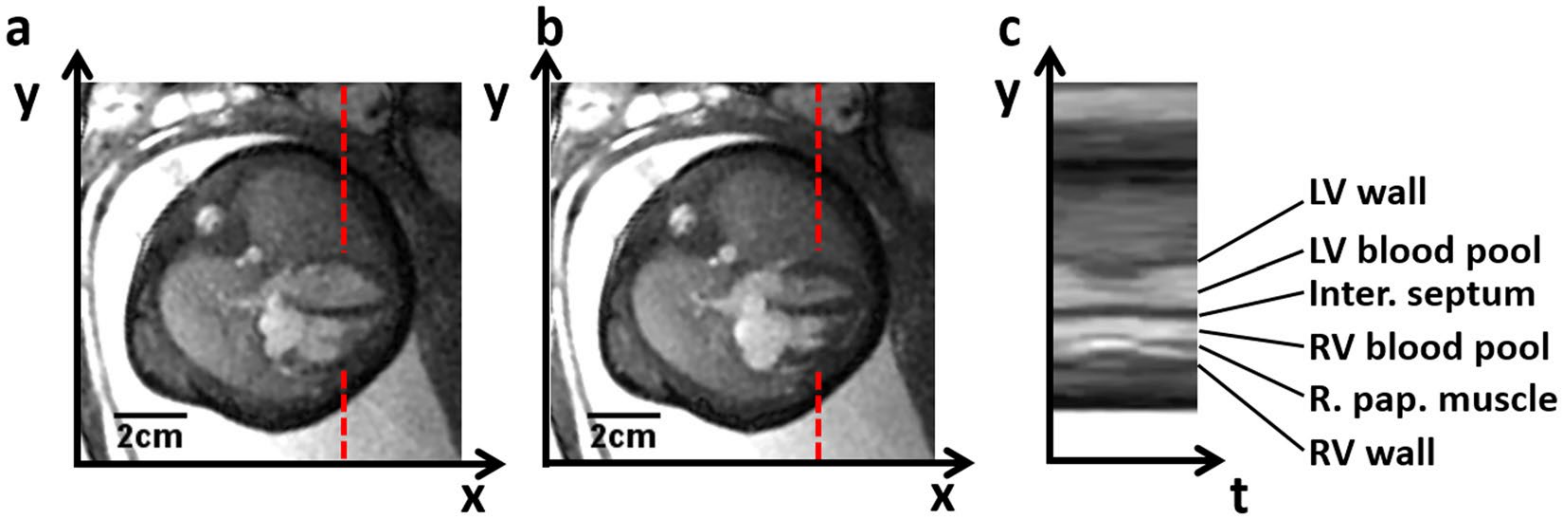
Self retro-gated reconstruction in a 32-week-old fetus in the 4-chamber view. The diastolic (**a**) and systolic (**b**) phases are presented, along with a y–t space trace (**c**) measured in the middle of the ventricles. The contraction of the myocardium and the thickening of the cardiac wall can be seen in the corresponding movie (Supplementary Information).

**Figure 4:**
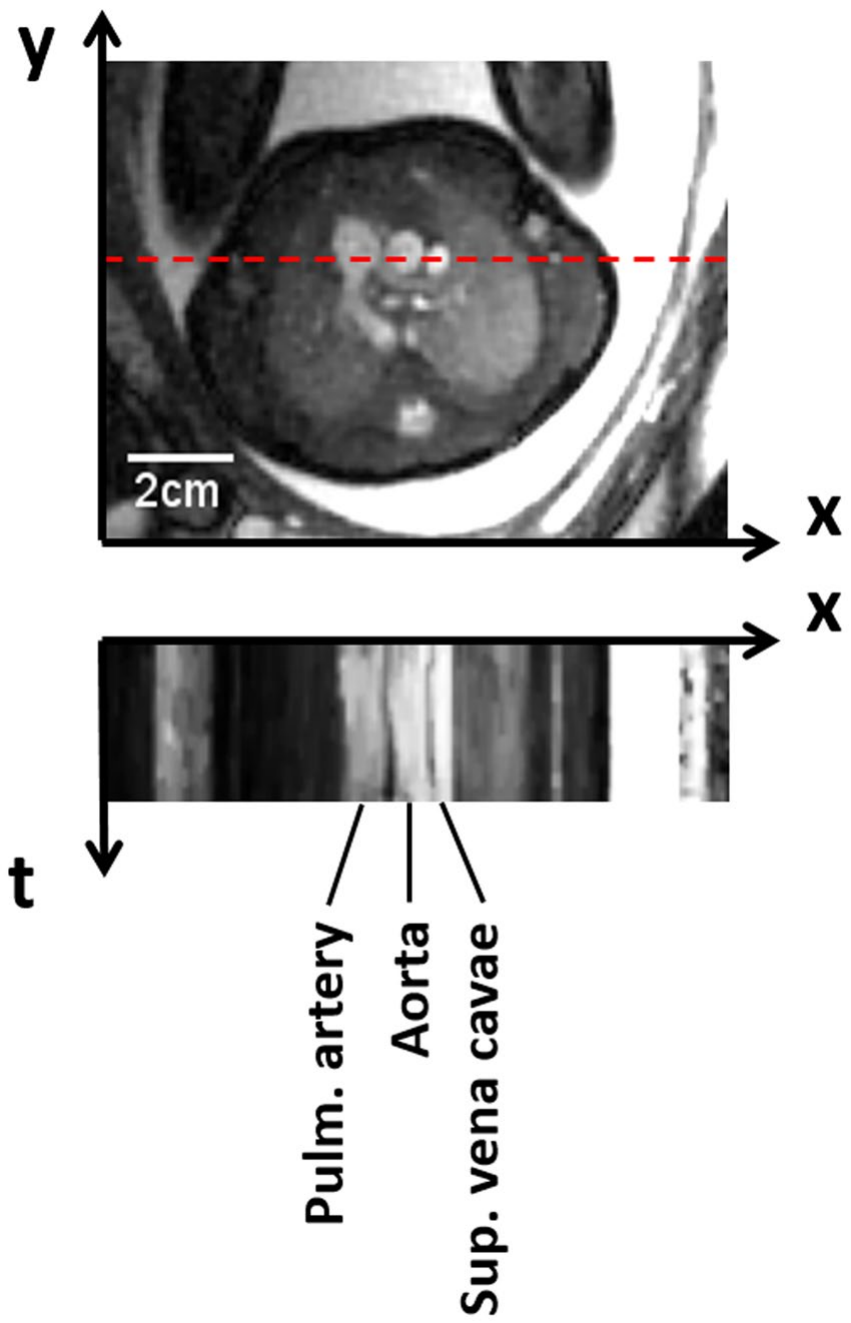
Self retro-gated reconstruction in a 32-week-old fetus in the 3-vessel view. From left to right at the level of the dashed line are the main pulmonary artery, the ascending aorta and the superior vena cava. For the corresponding movie, see the Supplementary Information.

MRI acquisitions were successful for all three targeted views in all patients (Fig. 2 and Supplementary Fig. S1). Typically, self-gated cardiac signals were successfully extracted, though parts of some data sets had to be excluded from retro-gating due to fetal motion that compromised acquisition planning or gating-signal extraction. On average, 20.5 MR real-time cine images per patient were acquired (see Figs 3, 4 and Supplementary Fig. S1) and 14.8 self-retro-gated cine images were reconstructed per patient. Quantitative comparisons were successfully performed in five of the six patients. In the first patient, the valve annuli and the endocardial border of the left ventricle could not be identified on the MR images due to fetal motion (Table 1). Therefore, no quantitative measurements were performed for this patient. In another patient, echocardiographic images could not be used for quantitative measurements due to incompatible data formatting, but the above-specified anatomical structures (items) could be identified and MR measurements were still included in the inter- and intra-observer analyses.

**Table 1:** Number of identified anatomical structures in each patient.

Patient (weeks of gestation at MRI examination)	Anatomical structures identified on MR images		Anatomical structures identified on echocardiographic images	
	Observer 1	Observer 2	Observer 1	Observer 2
1 (26 weeks 6 days)	6	7	13	12
2 (31 weeks 5 days)	13	13	13	13
3 (28 weeks 6 days)	12	12	13	12
4 (32 weeks 4 days)	13	13	13	13
5 (28 weeks 2 days)	13	13	13	13
6 (30 weeks 0 days)	13	12	13	12

On the MR cine images, Observer 1 identified 11.7 ± 2.8 items per patient (out of 13 items) and Observer 2 11.7 ± 2.3 items (Table 1), whereas on the echocardiographic images, they identified 13 ± 0 and 12.5 ± 0.5 items per patient, respectively. The discrepancy between the two modalities is solely due to poor image quality in the MR cine images of a single patient. The items that were most challenging to identify in the MR images were the LV papillary muscles (Table 2).

**Table 2:** Identified anatomical structures. Stars: difference between MRI and echocardiography, bold: difference between the two observers.

View	Anatomical structure	MRI (n = 6)		Echo (n = 6)	
		Observer 1	Observer 2	Observer 1	Observer 2
4-chamber view	Right atrium*	**5**	**6**	6	6
	Left atrium*	5	5	6	6
	Tricuspid valve annulus*	5	5	6	6
	Mitral valve annulus*	5	5	6	6
	Right ventricle	6	6	6	6
	Left ventricle	6	6	6	6
	Moderator band*	5	5	6	6
	Atrial septum*	5	5	**6**	**5**
	Ventricular septum	6	6	6	6
3-vessel view	Pulmonary artery	6	6	6	6
	Ascending aorta	6	6	6	6
	Superior vena cava*	**6**	**5**	**6**	**4**
Short-axis view	LV papillary muscles	4	4	6	6

Two fetuses had heart defects that were identifiable on MR images. One of them had a right-sided aortic arch and a second one a single left papillary muscle, as confirmed in both cases using the corresponding echocardiographic images.

**Figure 5:**
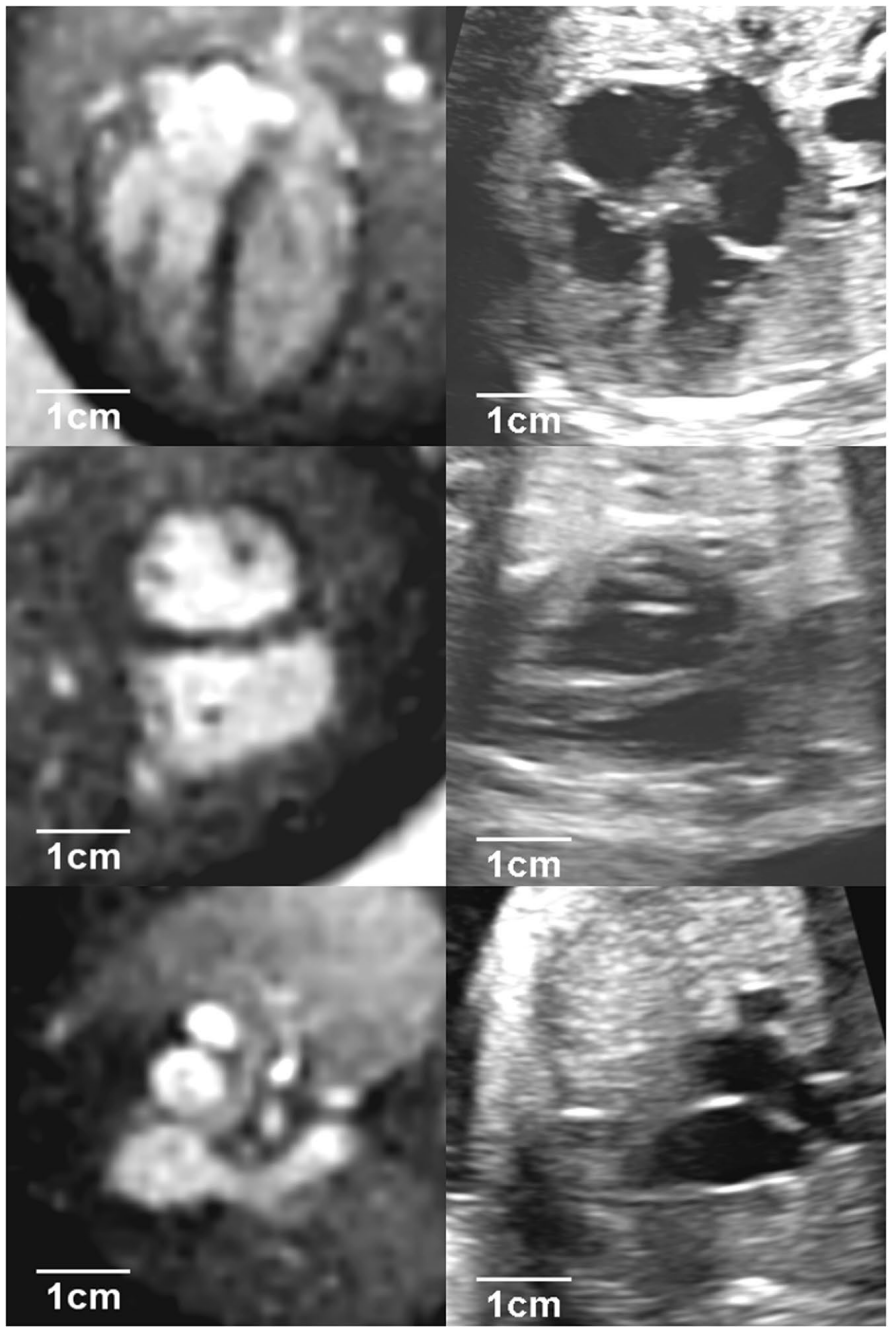
Comparison of MR (left) and echocardiographic (right) images. Top row: 4-chamber view; middle row: short-axis view; bottom row: 3-vessel view.

In the comparison between MR and echocardiographic images (Fig. 5), we found a good agreement for both observers for the diameter measurements (Table 3). Bland–Altman analyses yielded a bias of 0.30 mm and a 95% confidence interval (CI) of [−1.87;2.47] (p = 0.266) for Observer 1, and the linear regression (LR) was *y* = *0*.*81x* + *1*.*73* (r² = 0.625). For Observer 2, a bias of −0.50 mm (CI: [−2.71;1.71], p = 0.077), LR: *y* = *0*.*86x* + *0*.*57* (r² = 0.731) (Supplementary Fig. S2) was found.

**Table 3:** Quantitative measurements comparison.

			Bland–Altman			Linear Regression	
			Bias	CI	p	Model	r^2^
MR vs Echo	Diameter (mm)	Obs 1	0.30	[−1.87; 2.47]	0.266	y = 0.81x + 1.73	0.625
		Obs 2	−0.50	[−2.71; 1.71]	0.077	y = 0.86x + 0.57	0.731
	Area (mm²)	Obs 1	−2.11	[−46.17; 41.94]	0.798	y = 1.05x − 8.03	0.838
		Obs 2	9.21	[−66.59; 85.01]	0.522	y = 0.73x + 44.50	0.524
Inter observer	Diameter (mm)	MRI	−0.44	[−2.39; 1.51]	0.050	y = 1.00x − 0.45	0.748
		Echo	0.30	[−2.24; 2.84]	0.313	y = 0.92x + 0.90	0.576
	Area (mm²)	MRI	25.68	[0.01; 51.35]	<0.001	y = 0.928x + 34.29	0.941
		Echo	10.41	[−49.52; 70.34]	0.310	y = 0.86x + 27.05	0.653
Intra observer	Diameter (mm)	MRI	−0.22	[−2.19; 1.74]	0.310	y = 1.03x − 0.46	0.757
		Echo	0.21	[−1.59; 2.00]	0.329	y = 0.97x + 0.46	0.750
	Area (mm²)	MRI	2.06	[−8.22; 12.34]	0.245	y = 1.01x + 1.01	0.991
		Echo	7.83	[−35.48; 51.14]	0.291	y = 0.85x + 25.78	0.789

Area measurements for both modalities showed moderate to good agreement depending on the observer. Although the biases were of the same order, confidence intervals were almost twice as large for Observer 2 as they were for Observer 1. Bland–Altman analyses yielded a bias of −2.11 mm² (CI: [−46.17;41.94], p = 0.798), LR: *y* = *1*.*05x* − *8*.*03* (r² = 0.838) for Observer 1 (Supplementary Fig. S2g), and 9.21 mm² (CI: [−66.59;85.01], p = 0.522), LR: *y* = *0*.*73x* + *44*.*50* (r² = 0.524) for Observer 2 (Supplementary Fig. S2h).

## Inter-observer comparisons

The quality of the diameter measurements with MRI were similar for both observers, although a consistent offset was identified (Table 3), with the Bland–Altman analysis showing a bias of −0.44 mm (CI: [−2.39;1.51], p = 0.050), LR: *y* = *1*.*00x* − *0*.*45* (r² = 0.748) (Supplementary Fig. S3). By comparison, diameter measurements with echocardiography yielded a similar bias between the observers, 0.30 mm (CI: [−2.24;2.84], p = 0.313), whereas the correlation was lower than for MR measurements, LR: *y* = *0*.*92x* + *0*.*90* (r² = 0.576).

For the area measurements with MRI, there was a significant bias between the two observers of 25.68 mm² (CI: [0.01;51.35], p < 0.001), LR: *y* = *0*.*928x* + *34*.*29* (r² = 0.941). By comparison, the bias in echocardiographic measurements was 10.41 mm² (CI: [−49.52;70.34], p = 0.310), LR: *y* = *0*.*86x* + *27*.*05* (r² = 0.653). Despite a low bias, the confidence interval for the echocardiographic measurements was twice as large as that obtained with MRI. As a consequence, the correlation coefficient between the observers was higher for MR measurements than for the echocardiographic ones (Supplementary Fig. S3).

## Intra-observer comparisons

Repeated diameter measurements with MRI showed good intra-observer reproducibility (Table 3 and Supplementary Fig. S4), with the Bland–Altman analysis yielding a bias of −0.22 mm (CI: [−2.19;1.74], p = 0.310), LR: *y* = *1*.*03x* − *0*.*46* (r² = 0.757). The reproducibility of echocardiographic measurements had similar bias (0.21 mm, CI: [−1.59;2.00], p = 0.329), and correlation (LR*: y* = *0*.*97x* + *0*.*46*, r² = 0.750).

The MRI-based area measurements showed high intra-observer reproducibility (Table 3 and Supplementary Fig. S4), with a bias of 2.06 mm² and a narrow CI: [−8.22;12.34] (p = 0.245), and a LR close to identity: *y* = *1*.*01x* + *1*.*01* (r² = 0.991). By comparison, the bias in the echocardiographic measurements was 7.83 mm² (CI: [−35.48;51.14], p = 0.291), LR: *y* = *0*.*85x* + *25*.*78* (r² = 0.789). The apparent lower reproducibility of echocardiographic measurements is essentially due to one outlier. When this outlier was excluded from the analyses, the bias decreased to 1.42 mm² (CI: [−16.90;19.75], p = 0.66), LR: *y* = *1*.*00x* + *1*.*57* (r² = 0.962), which approaches the reproducibility obtained with MRI.

## Discussion

A clear majority of anatomical structures of the fetal heart that are commonly identified during routine clinical fetal echocardiography were visible in the MR images, although fetal motion may still affect the success rate. The quantitative comparison of MR and echocardiographic images showed good overall agreement. The variability between the two modalities can be explained by how the examinations are performed. Fetal echocardiography is a real-time examination, where the imaging plane can be continually adjusted, while MR is a ‘single-shot’ type of examination, where the imaging plane is predefined and mandates absence of fetal motion during the acquisition. Therefore, the measurements were likely performed in slightly different planes in the MR and echocardiographic images, respectively, and this may explain some of the observed inconsistencies between MRI and the gold standard.

Moreover, the fetal echocardiograms contained significantly more images per patient, increasing the likelihood that the two observers did not use the exact same images for their measurements. This may partly explain the increased inter-observer variability of echocardiographic measurements when compared to MR.

Despite the high quality of the reconstructed MR images, improvements including automated ROI selection to derive the cardiac motion signal will have to be implemented to improve the ease-of-use and to minimize operator dependency. The proposed strategy currently relies heavily on the operator to judiciously select the ROI to perform the cross-correlation. The Pearson correlation further needs a sufficient number of pixels with a significant signal-intensity change during the cardiac cycle to reliably detect the heart motion and to generate a good gating signal. Such signal extraction in short axis views is straightforward, given the relatively high number of pixels with considerable signal intensity change during the cardiac cycle of the fetus. However, this is more challenging in the four-chamber and three-vessel views, where the number of pixels with substantial signal variation is reduced. This highlights the importance of the ROI placement to obtain the best gating signal.

Once this cross-correlation or gating signal is extracted, the operator selects the trigger points. In adults, we initially proposed to select an end-systolic image to be cross-correlated with the remaining real-time cine frames to automatically detect the signal peaks^25^. In fetuses, however, the small size of the heart and the high degree of undersampling required to achieve high temporal resolution led to noise and streaking artifacts in the real-time images, precluding an automated detection of the trigger points. Therefore, a multitude of avenues to improve this trigger signal have been explored in this study: selecting the frame of reference in diastole instead of systole, performing the cross-correlation on the signal phase instead of the magnitude, or selecting the triggers in the valleys of the cross-correlation signal instead of the peaks. Further developments similar to those proposed by van Ameron *et al*.^28^ may lead to a better gating signal by analyzing the variation frequency within the defined ROI.

The Metric Optimized Gating approach developed by Roy *et al*.^24^ does not require the user to manually select the trigger as it aims at automatically decreasing the entropy in the images by iteratively improving the data consistency. This is achieved by sorting the data in cardiac phases and measuring the artifacts generated by the cardiac motion. This approach, however, requires a careful manual selection of the ROI to measure the entropy reliably. A new development that consists in a combination of the Metric Optimized Gated approach with compressed sensing led to a decrease of the scan time that is similar as that reported here^29^. The image quality between the two techniques is comparable, but further comparisons need to be performed, especially in views other than the short-axis.

Fetal movement during data collection mandated that some images had to be re-acquired. However, in the case of motion during a short period, the motion-corrupted readouts were simply excluded from the retro-gated reconstruction, with little adverse effect on image quality. This is due to the properties of the golden-angle sampling, providing images of decent quality even under non-optimal conditions. Moreover, a quick assessment of fetal bulk motion can be performed on the scanner by reconstructing images in real time during data acquisition with a low temporal resolution. Such a feedback enables well-informed decision making regarding a potential reacquisition or modification of the scan plane, and can be easily combined with future interactive planning to adapt the imaging plane in real time^30^.

The choice of acquiring three slices within a breath hold rather than one single slice reduces the amount of available *k*-space data per image, and is therefore detrimental to image quality. Having fewer radial profiles leads to a lower flexibility in selecting motion-free data and increases both artifacts and noise in the images. However, acquiring only one slice per breath hold is not optimal for fetal cardiac imaging. First, fetal motion may cause the anatomy of interest to move outside of the imaging plane. Therefore, acquiring a stack of slices increases the likelihood of successful imaging of the heart despite fetal bulk motion. Second, intracardiac and vascular anatomy are complex 3D structures, which are difficult to assess from a single 2D slice. In this study, most of the fetuses did not have heart defects, although our approach is designed to scan complex congenital heart disease. By acquiring a stack of images, we can obtain a more detailed 3D representation of the anatomy and adequately evaluate the anatomical relationships between cardiac connections and blood vessels. As for the reconstruction regularization parameters, they were carefully optimized for the field strength and imaging sequence parameter settings utilized in this report. Should any of these boundary conditions change, it is anticipated that regularization parameters may have to be adjusted.

Measurements of LV systolic function were performed by calculating the fractional area change in the mid-ventricular short-axis view. Although this is not a validated measure of LV systolic function, it shows that LV end-diastolic and end-systolic volumes, and therefore ejection fraction, could be measured by MRI in fetuses. In addition, the reproducibility of MR measurements was very high and significantly higher than that of echocardiography. Finally, despite the patients included in this study being referred for non-cardiac fetal scans, heart defects were identified with both modalities in two fetuses.

These preliminary results are encouraging and promise a reliable second line imaging modality for patients in whom echocardiography is inconclusive. However, further developments are needed to enable fetal cardiac MR examination during the second trimester of pregnancy to better inform clinicians and patients. The current possibilities with MRI are not optimal in that regard, since earlier examinations imply smaller fetal structures and more movements of the fetus. In addition, future developments towards flow measurements in the great vessels, which provides important information, are mandatory in order to provide clinicians with a complete MRI toolbox to assess the fetus’ condition when echocardiography is not conclusive.

## Methods

### Magnetic resonance imaging

#### Ethics statement

All methods were performed in accordance with the relevant guidelines and regulations. The study was performed with the approval of the Commission cantonale d’éthique de la recherche sur l’être humain, and written informed consent was obtained from all patients prior to data acquisition.

#### Image acquisition

MRI data were acquired on a 1.5 T clinical MR scanner (MAGNETOM Aera, Siemens AG, Healthcare Sector, Erlangen, Germany) with an 18-channel body array coil and a 32-channel spine coil for signal reception. Imaging was performed with a 2D slice-selective untriggered continuous balanced steady-state free precession sequence that was modified to acquire radial readouts with a golden-angle trajectory. The parameters were: field-of-view = 260 × 260 mm², matrix size = 256 × 256 pixels, pixel size = 1.0 × 1.0 mm², slice thickness = 4.0 mm, TE/TR = 1.99/4.1 ms, RF excitation angle = 70°, slices = 3, shot per slice = 1, radial readouts per slice = 1600, acquisition time per slice = 6.7 s and bandwidth = 1028 Hz/pixel. The real-time images used for the data-driven self-gating procedure were reconstructed from 15 radial readouts, corresponding to a window width of 61.5 ms. The reconstructed temporal resolution of the real-time images was improved through a 70% view-sharing approach incorporated into the reconstruction, resulting in a 18.5-ms temporal resolution.

#### Image reconstruction

The reconstruction of the real-time images was performed off-line with an in-house MATLAB (MathWorks, Natick, Massachusetts, USA) script implementing a *k*-*t* sparse SENSE algorithm model^31^, with wavelet and total variation for spatial and temporal regularizations respectively, described by the equation

1 $$arg\,mi{n}_{m}{\Vert FCm-s\Vert }_{2}^{2}+{\lambda }_{1}{\Vert {\rm{\Psi }}m\Vert }_{1}+{\lambda }_{2}{\Vert {{\rm{\Delta }}}_{s}m\Vert }_{1}+{\lambda }_{3}{\Vert {{\rm{\Delta }}}_{t}m\Vert }_{1}$$

where **F** is the non-uniform fast Fourier operator^32^ defined on the golden-angle radial pattern, **C** the coil sensitivity in the x–y space, **m** the images to be reconstructed, **Ψ** a spatial 2D wavelet transform and **Δ**_**s**_ and **Δ**_**t**_ the discretized spatial and temporal finite-difference gradient operator for total variation regularization, respectively. The regularization parameters **λ**_**1–3**_ were selected empirically by visual inspection to find an appropriate tradeoff between data consistency and compression artifacts. For the real-time reconstruction these parameters were always set to (**λ**_**1**_ = 0.008, **λ**_**2**_ = 0.008, **λ**_**3**_ = 0.1). The nonlinear optimization problem defined by Equation [1] was solved with a conjugate gradient algorithm^33^ including a line-search method based on golden-section search and parabolic interpolation^34^. The density compensation was performed with a ramp filter applied to each readout. The coil sensitivities were computed from a fully sampled region at the center of *k*-space using an approach described by Pruessmann *et al*.^35^. For the reconstruction of the final, self-retro-gated images during the second pass (Fig. 1d), the regularization parameters were empirically adapted and remained constant for each patient as well (**λ**_**1**_ = 0.004, **λ**_**2**_ = 0.004, **λ**_**3**_ = 0.05). When compared to the above values that were used for reconstruction of the real-time images, these regularization parameter values can be reduced because of the lower undersampling of k-space.

### Ultrasonography

Ultrasound examinations were performed on Voluson E8 Expert and Voluson E10 devices (GE Healthcare, Glattbrugg, Switzerland) with the RM6C 3D/4D curved array transducer with a frequency range of 1.0–7.0 MHz.

## Electronic supplementary material

Figure S1

Figure S2-4
