# Author Correction: Fetal cardiac cine magnetic resonance imaging *in utero*

**DOI:** 10.1038/s41598-018-25806-w

**Published:** 2018-05-15

**Authors:** Jerome Chaptinel, Jerome Yerly, Yvan Mivelaz, Milan Prsa, Leonor Alamo, Yvan Vial, Gregoire Berchier, Chantal Rohner, François Gudinchet, Matthias Stuber

**Affiliations:** 1 Department of Radiology, University Hospital (CHUV) and University of Lausanne (UNIL), Lausanne, Switzerland; 2 Center for Biomedical Imaging (CIBM), Lausanne, Switzerland; 3 Division of Pediatric Cardiology, Department Woman-Mother-Child, University Hospital (CHUV) and University of Lausanne (UNIL), Lausanne, Switzerland; 4 Division of Obstetrics and Gynecology, Department Woman-Mother-Child, University Hospital (CHUV) and University of Lausanne (UNIL), Lausanne, Switzerland

Correction to: *Scientific Reports* 10.1038/s41598-017-15701-1, published online 14 November 2017

This Article contains errors in Reference 28, which is incorrectly given as:

‘van Ameron, J. *et al*. Fetal Cardiac Cine Imaging From Motion-Corrected Super-Resolution Reconstruction of Highly-Accelerated Real-Time MRI. *Proceedings of the International Society for Magnetic Resonance in Medicine* **24**, 458 (2016).’

The correct reference is listed below as ref. 1.
