# Supplementary figures and images for: Fetal cardiac cine magnetic resonance imaging in utero
Source: Sci Rep. 2017 Nov 14;7:15540. doi: 10.1038/s41598-017-15701-1 (PMC5686109; doi:10.1038/s41598-017-15701-1)

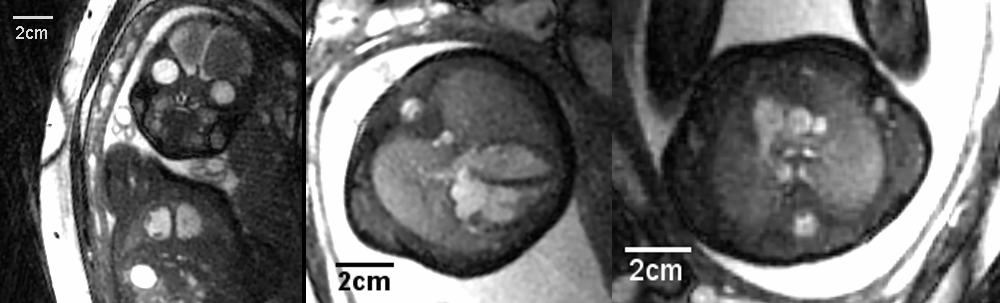

Supplement: Supplementary file 1 — Figure S1 [file 41598_2017_15701_MOESM1_ESM.gif]
