# Supplementary material for: Fetal cardiac cine magnetic resonance imaging in utero
Source: Sci Rep. 2017 Nov 14;7:15540. doi: 10.1038/s41598-017-15701-1 (PMC5686109; doi:10.1038/s41598-017-15701-1)
Supplement: Supplementary file 2 — Figure S2-4 [file 41598_2017_15701_MOESM2_ESM.pdf]

Fetal cardiac cine magnetic resonance imaging *in utero*

Jerome Chaptinel[1], Jerome Yerly[1,2], Yvan Mivelaz[3], Milan Prsa[3], Leonor Alamo[1], Yvan Vial[4], Gregoire Berchier[1], Chantal Rohner[1], François Gudinchet[1,†], Matthias Stuber[1,2*]

[1]*Department of Radiology, University Hospital (CHUV) and University of Lausanne (UNIL), Lausanne, Switzerland,*

[2]*Center for Biomedical Imaging (CIBM), Lausanne, Switzerland,*

[3]*Division of Pediatric Cardiology, Department Woman-Mother-Child, University Hospital (CHUV) and University of Lausanne (UNIL), Lausanne, Switzerland,*

[4]*Division of Obstetrics and Gynecology, Department Woman-Mother-Child, University Hospital (CHUV) and University of Lausanne (UNIL), Lausanne, Switzerland*

[†] *Deceased 3 August 2016.*

Correspondence to: Prof. Matthias Stuber (Matthias.stuber@chuv.ch)
Center for BioMedical Imaging (CIBM)
Centre Hospitalier Universitaire Vaudois (CHUV)
Rue de Bugnon 46, BH 8.80
1011 Lausanne, Switzerland

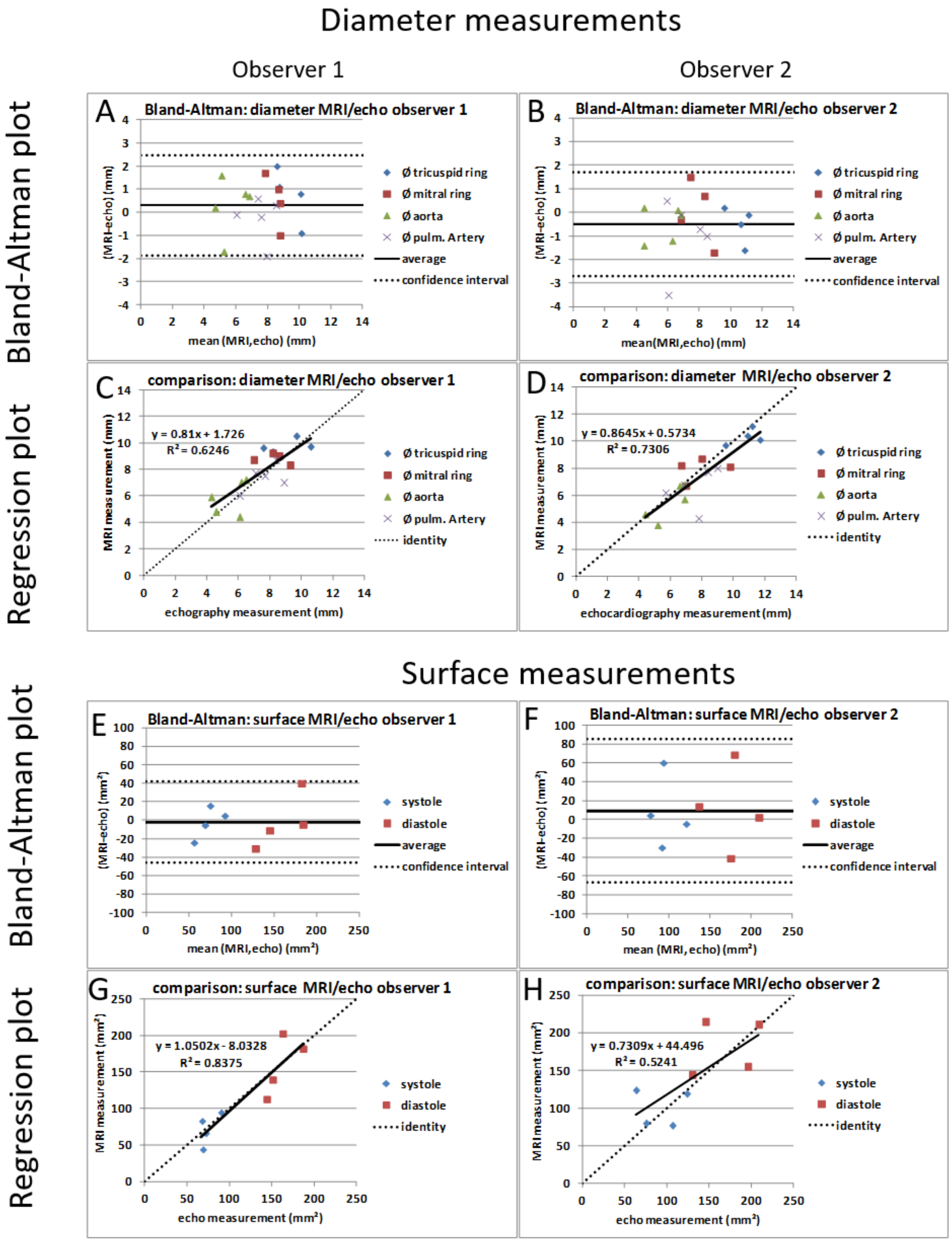

Figure S2: Comparison of diameter (ABCD) and area (EFGH) measurements performed on MR and echocardiographic images for the two observers.

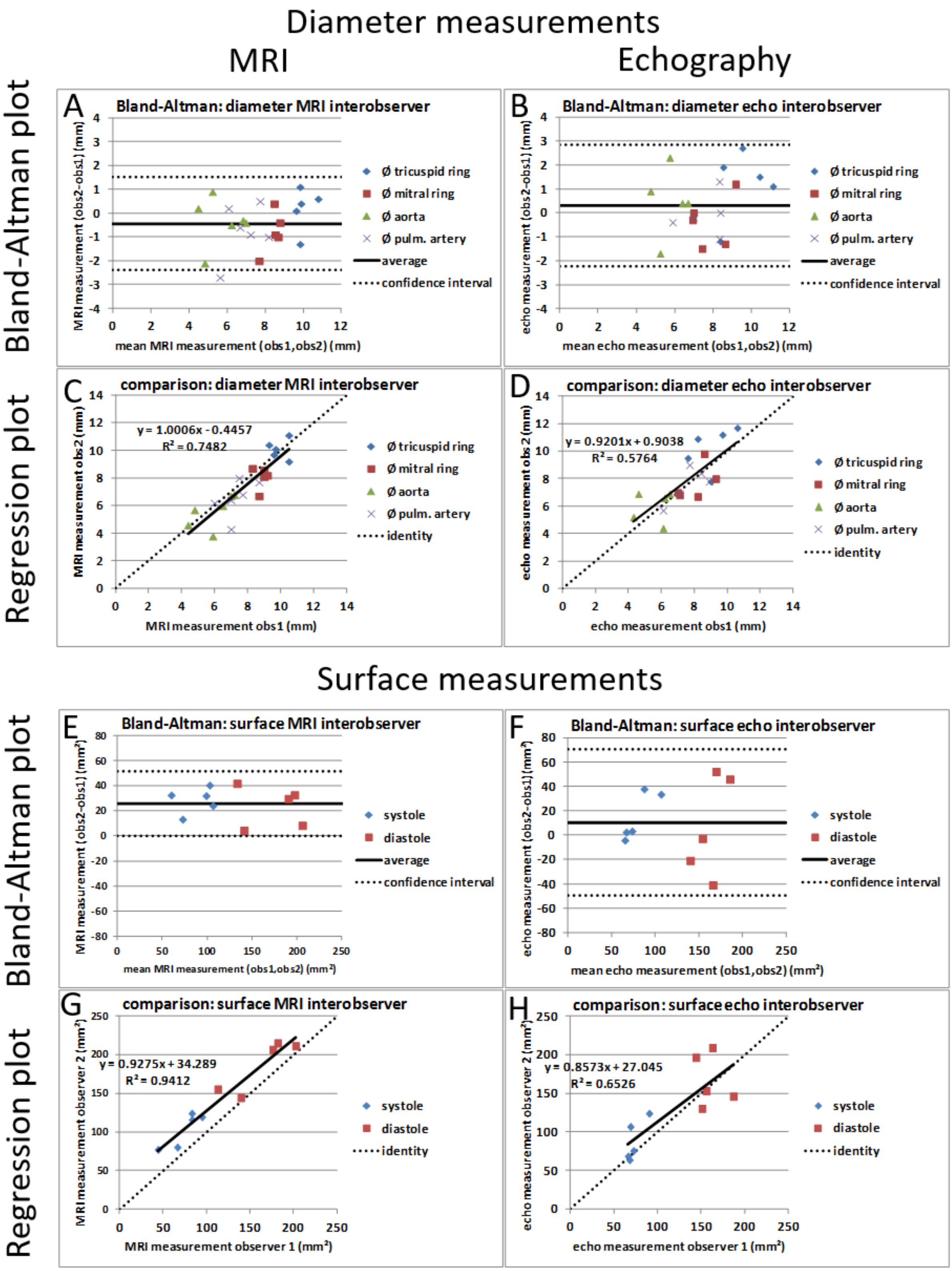

Figure S3: Inter-observer analysis for diameter (ABCD) and area (EFGH) measurements for MR (left column) and echocardiographic (right column) images.

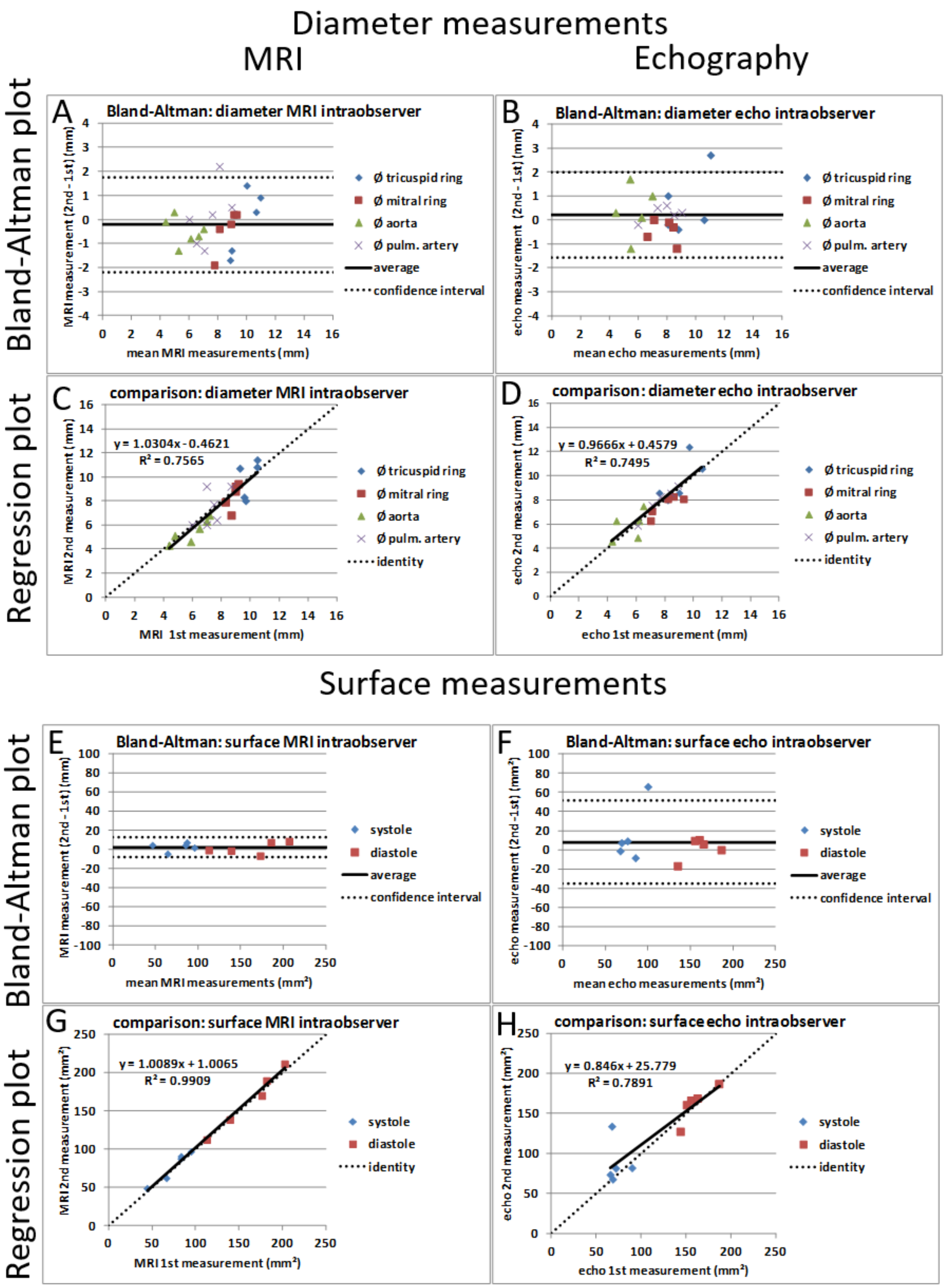

Figure S4: Intra-observer reproducibility for Observer 1 for diameter (ABCD) and area (EFGH) measurements in MR (left column) and echocardiographic (right column) images.
